# Adipose-derived mesenchymal stem cells and wound healing: Potential clinical applications in wound repair

**DOI:** 10.15537/smj.2022.43.10.20220522

**Published:** 2022-10

**Authors:** Sami G. Almalki

**Affiliations:** *From the Department of Medical Laboratory Sciences, College of Applied Medical Sciences, Majmaah University, Majmaah, Kingdom of Saudi Arabia*.

**Keywords:** cutaneous wound healing, adipose-derived mesenchymal stem cells, epithelial cell differentiation, angiogenesis, immunomodulation, inflammation, proliferation, maturation and remodeling

## Abstract

Delayed and chronic wounds result from the dysregulation of molecular and cellular events associated with wound healing, including migration, inflammation, angiogenesis, extracellular matrix (ECM) remodeling, and re-epithelialization. Adipose tissue is an abundant, easily accessible, and rich source of mesenchymal stem cells (MSCs) with high therapeutic potential. In addition to their capability to differentiate into various lineages with specialized functions, adipose-derived MSCs (AMSCs) can mediate to the wound repair process through the secretion of different growth factors and mediators rather than making structural contribution alone. Adipose-derived MSCs mediate the formation of blood vessels, recruit progenitor cells, stimulate cell differentiation and ECM formation, and promote wound healing by releasing immune mediators and exosomes. Herein, we discuss and review the therapeutic potential of AMSCs for wound repair via acceleration of wound closure, re-epithelialization, enhancement of angiogenesis and immunomodulation of prolonged inflammatory responses, as well as the current challenges in clinical implementation.

**C**hronic cutaneous wounds are injuries that can extend to the subcutaneous level, but do not affect bones. In the United States of America, approximately 6 million Americans experience chronic wounds.^1^ Chronic cutaneous wounds that result from burns, traumatic injuries, and diabetes, are serious healthcare burden worldwide and a major cause of morbidity.^2^ Healthcare treatment and hospitalization costs approximately 25 billion each year in the United States.^3^ At the molecular level, wound healing eventuates in 3 distinct steps, the inflammatory, proliferative, and maturational (remodeling) phases.^4^ These phases are influenced by different host factors, external factors and wound characteristics, and prolonged wound healing is more likely to develop into scars.^5^ Although there has been a considerable progress in chronic wound treatment, including the application of bioengineered skin equivalents and growth factors, the current treatment strategies are limited and only partially effective for non-healing and chronic wounds.^4,6^ Therefore, A curative strategy for the treatment of chronic cutaneous wounds is needed to improve patient outcomes.

Recently, mesenchymal stem cells (MSCs) have been reported as a tool for promising cell-based therapy and shown to play key roles in tissue repair and regeneration.^1^ Mesenchymal stem cells are attractive candidates in regenerative medicine because of their migratory ability, high proliferative rate, ease of access, and immunomodulatory effects.^7^ They have the capability to differentiate into several lineages.^8^ They are characterized by a fibroblast-like morphology (the ability to self-renew; the expression of CD markers such as CD29, CD44, CD73, CD90, and CD105; and a lack of hematopoietic and macrophage markers such as CD11, CD14, CD34, and CD45).^9-11^ Adult MSCs can be extracted from various sources, including the umbilical cord blood, placental tissue, adipose tissue, peripheral blood, lung, liver, heart, bone marrow (BM), testes, pancreas, spleen, and dental pulp.^12-14^ Among the different types of MSCs, bone marrow-derived MSCs (BM-MSCs) and adipose-derived MSCs (AMSCs) are the most frequently used cells for tissue repair and skin regeneration in the treatment of non-healing wounds. Even though some clinical studies of BM-MSCs have reported issues its use as an MSC source, including the extremely low density of MSCs, morbidity, and invasive isolation procedures, the BM is the first and most commonly used source of MSCs in research.^1,12,15^ Adipose tissue is an easily accessible, abundant, and rich source of MSCs with a great therapeutic potential.^16^ The high MSC yield from adipose tissue (0.01% with 95% purity) compared with that from BM makes AMSCs an appealing cell source for the repair of cutaneous wounds and skin regeneration.^16,17^ Adult AMSCs have been studied as an attractive alternative to BM-MSCs.

Multiple new strategies for the treatment of chronic cutaneous wounds are being investigated in clinical trials. The use of MSC-based regenerative therapy for the treatment of chronic cutaneous wounds has been rising over the last 2 decades. Bone marrow-derived MSCs and ASMCs represent the most recent advancements in the treatment of chronic wounds.^18^ Patients with chronic wounds are MSC-deficient, and the patient-derived MSCs may provide potential therapeutic strategies to overcome this deficiency.^19^ Delayed and chronic wounds result from the dysregulation of molecular and cellular events associated with wound healing, including migration, inflammation, angiogenesis, extracellular matrix (ECM) remodeling, and re-epithelialization.^20^ Mesenchymal stem cells play a crucial role in these events. Various studies have demonstrated that the administration of MSCs, not only mediates cutaneous wound repair, but also augment the healing of acute and chronic wounds.^21,22^ This study aimed to review and discuss the potential therapeutic contributions of AMSCs in cutaneous wound healing and the current challenges in their clinical implementation.

## Physiology of cutaneous wound healing

**Figure 1:**
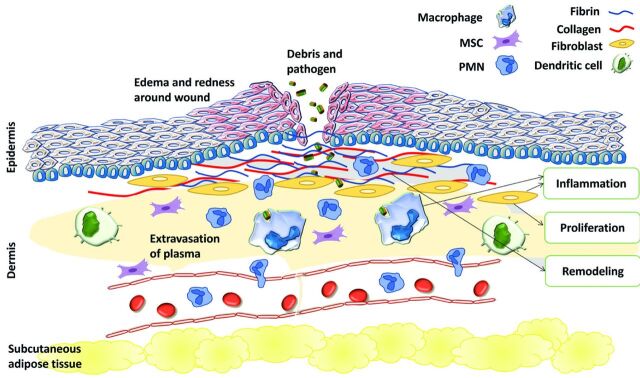
- The cascade of events after a superficial skin trauma. MSC: Mesenchymal stem cell, PMN: polymorphonuclear leukocytes

The wound healing process initiates several molecular and cellular events that involve different cellular compartments of the skin and ECM. The skin consists of 2 layers: the epidermis, which is the superficial layer, and the dermis, which is the second layer in the skin.^23^ The epidermis consists of epithelial cells, and functions as a biochemical and physical barrier to protect the underlying layer from infections and dehydration.^24^ The dermis, which is composed of fat and connective tissue, maintains body temperature, supplies nutrients to the epidermis via blood, and gives the skin its elasticity and thickness.^24^ During cutaneous wound healing, different cell types are involved in restoration of the barrier functions of skin and regeneration of the damaged skin. These cells undergo several biological events, including proliferation, migration, and differentiation, to regenerate new skin (Figure 1).^25^ Under normal physiological conditions, the acute cutaneous wound healing process is characterized by 3 distinct stages of repair: inflammation, proliferation, and tissue remodeling.^25^ Failure to proceed through an orderly process or dysregulation of one of these phases results in chronic (non-healing) wounds or scars.^26^

## Inflammation

Skin injury immediately results in hemostasis, which involves vascular constriction and blood clotting cascade to prevent blood loss.^5^ The fibrin clot and injured tissue release growth factors and pro-inflammatory cytokines into the local wound site to recruit inflammatory cells.^5^ The release of cytokines promotes the migration of inflammatory cells, such as neutrophils and monocytes, into the injured tissue and initiates the inflammatory phase.^25,26^ Inflammation begins upon injury and lasts for 4-6 days.^27^ The recruited neutrophils and macrophages prevent bacterial infection and remove cellular debris at the wound site via phagocytosis. It has been reported that elevated infiltration of neutrophils and macrophages leads to delayed healing and the deregulation of interleukin-1β (IL-1β) and tumor necrosis factor-α (TNF-α).^28,29^ Chronic wounds are prolonged inflammation that fails to proceed, and involves high expression of IL-1β and TNF-α.^26^ In addition to phagocytosis, macrophages initiate the transition to the proliferative phase through the release of transforming growth factor-β (TGF-β), ILs, and TNF-α, which promote the migration of fibroblasts, endothelial cells, and keratinocytes to the wound site.^29,30^

## Proliferation

This phase comprises several biological processes, including granulation, angiogenesis, collagen deposition, and epithelialization.^18,29^ The proliferative phase begins immediately after the inflammatory phase and lasts for up to 10 days.^29^ During this phase, keratinocytes proliferate and migrate to the wound site for re-epithelialization. Keratinocytes also synthesize proteins to restore the basement membrane.^31^ Several growth factors, cytokines, and nitric oxide, which are released by macrophages, induce the re-epithelialization process.^29^ Angiogenesis, which is characterized by the formation of new blood vessels, is promoted by various growth factors including vascular endothelial growth factor (VEGF), platelet-derived growth factor (PDGF), and fibroblast growth factor (FGF).^29^ These growth factors stimulate the proliferation and migration of endothelial cells into angiogenic signaling site. The formation of new blood vessels is an early process during the proliferative phase, which provides nutrients and growth factors for granulation and tissue deposition.^29,32^ During the granulation process, the migrated fibroblasts proliferate and synthesize collagen type III, glycosaminoglycans, and fibronectin (ECM components) to restore the dermis, in response to PDGF, TGF-β, and FGF expression.^5,27^ The migration of fibroblasts from surrounding tissues into the wound site is facilitated by the production of matrix metalloproteinases (MMPs).^29^ Fibroblasts can also be generated from differentiated BM-MSCs during the long-wound healing process.^33^ The migrated fibroblasts can transform into myofibroblasts that contract to close the wound gap.^33,34^

## Maturation and remodeling

Following the buildup of ECM and gap closure at the wound site, the remodeling phase begins which may last for weeks or years. The major role of this phase is to reorganize the deposition of collagen into a well-arranged network.^29^ The reorganization process involves substitution of collagen III with collagen I fibers.^35^ During this process, MMPs play key roles in ECM remodeling through their degradative function. Myofibroblasts and fibroblasts at the wound site undergo apoptosis, resulting in the formation of avascular and acellular tissues.^29,32^ Failure of myofibroblasts to undergo apoptosis results in the pathophysiological processes of wound healing and hypertrophic scar formation.^29^ Serious injuries may result in the inability of healing skin to restore hair follicles and sweat glands.^36^

## Pathophysiology of wound healing

Multiple factors that influence the wound healing process are classified into local and systemic factors. Local factors influence the characteristics of the wound and impair the healing process, such as ischemic tissues, foreign bodies, contamination, bacterial infection, and elevated tissue pressure.^5,27^ Systemic factors are the overall health parameters of patients that influence the wound healing process; these include age, gender hormones, diabetes, stress, obesity, hypothermia, smoking, and nutrition. All these factors can lead to the failure of progression of wound repair process through normal stages of wound healing, resulting in a chronic wound.^5,27^ Excessive inflammation during cutaneous wound repair has been linked to chronic wounds and scar formation.^31^ In a persistent inflammatory state, macrophages and neutrophils continuously release large amounts of proinflammatory cytokines which upregulate the expression of MMPs.^29,37^ The high expression of MMPs at the wound site results in the degradation or inactivation of various important factors for wound repair, including growth factors and wound matrix.^37^ In pathophysiological conditions, the restoration of deep dermal layers is not efficient, which leads to either chronic wound or scar formation with loss of tissue function and structure.^26^ The treatment of chronic wounds is challenging because of our weak understanding of the underlying pathological mechanisms. Therefore, new therapeutic strategies for the treatment of chronic wounds are desired.

## Mesenchymal stem cell-based therapy and wound healing

**Figure 2:**
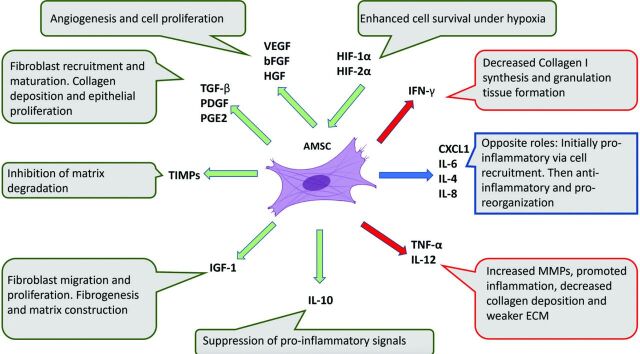
- Major cytokines and growth factors released by mesenchymal stem cells and their role in wound healing and cutaneous regeneration. AMSC: adipose-derived mesenchymal stem cell, VEGF: vascular endothelial growth factor, bFGF: basic fibroblast growth factor, HGF: hepatocyte growth factor, TGF-β: transforming growth factor-β, PDGF: platelet-derived growth factor, PGE2: prostaglandin E2, TIMPs: tissue inhibitors of metalloproteinases, IGF-1: insulin-like growth factor 1, IL: interleukin, TNF-α: tumor necrosis factor-α, CXCL1: the chemokine (C-X-C motif) ligand 1, IFN-γ: interferon gamma, HIF-1α: hypoxia-inducible factor 1 alpha

The use of stem cells for the treatment of chronic wounds has primarily focused on MSCs, also known as adult stem cells. Adipose tissue provides an easily accessible source of MSCs that could potentially avoid the unethical issues related to the use embryonic stem cells. Among the different sources of MSCs that might have therapeutic potential for chronic wound repair and injured skin regeneration, AMSCs have been administered structurally and systemically owing to their crucial role in cutaneous wound repair.^33^ In addition to their capability to differentiate into various cell types with specialized functions, AMSCs can mediate the wound healing process by secreting different growth factors and mediators rather than making just structural contributions.^33^ Multiple studies have shown that AMSCs can boost the repair process of chronic cutaneous wounds when applied locally to the injured skin in patients.^33,38^ Through the release of growth factors, cytokines, and other mediators, AMSCs mediate the formation of new blood vessels, recruit progenitor cells, stimulate cell differentiation, and induce ECM formation (Figure 2).^33^ Moreover, AMSCs have immunomodulatory properties that can regulate fibrosis and inflammation and promote wound healing through the secretion of immune mediators such as interferon-λ, TNF-α, IL-1α, IL-1β, and prostaglandin E 2.^33,39^ A hypoxic wound microenvironment induces the expression of hypoxia-inducible factors 1a and 2a. These molecules upregulate the expression of VEGF, hepatocyte growth factor, and bFGF in MSCs resulting in an augmented repair response in the wound microenvironment.^40^ As previously reported, MSCs can express tissue inhibitors of matrix metalloproteinases which gradually inhibit all active matrix metalloproteinases, thereby limiting the destruction of wound microenvironment to trauma-generated debris.^41^ This effect enhances the deposition of a new matrix.^33^ In the following subsections, we will discuss and review the potential therapeutic strategies based on AMSCs for cutaneous wound repair via enhancement of wound closure, re-epithelialization, angiogenesis, and immunomodulation.

## Contribution of AMSCs to cutaneous wound healing

The formation of new blood vessels is promoted by different growth factors including VEGF. Adipose-derived MSCs on the wounds show site-specific differentiation to epithelial and endothelial cells, which suggest that AMSCs have the capacity to enhance cutaneous wound healing through differentiation, vasculogenesis and secretion of the major angiogenic factor (VEGF).^33^ It has been suggested that AMSCs accelerate wound healing by releasing the growth factors that promote angiogenesis.^42^ Zografou et al^43^ demonstrated that AMSCs increase the survival of skin grafts and show higher collagen density and VEGF expression which mediate proliferation.

Adipose-derived MSCs enhance wound healing via the mediation of several processes, including proliferation, migration, and angiogenesis at the wound site.^44^ The implantation of single-layer AMSC sheets and triple-layer AMSC sheets onto a full-thickness skin wound for 3 weeks resulted in a significantly smaller wound area and higher angiogenisity than those in the non-treated control.^45^ Autologous AMSC-based therapy is an ideal strategy for minimizing immune rejection. Autologous transplantation of AMSCs enhanced wound healing in a diabetic rat, and the results showed a significant increase in skin graft survival, angiogenesis, and epithelialization.^43^ Another study showed that implantation of autologous AMSCs containing an atelocollagen matrix with silicon membrane in full-thickness round skin defects significantly enhanced granulation and angiogenesis compared to those in mice treated with ACMS alone.^46^ In a murine model, a group of scientists investigated the effects of AMSCs, with an acellular dermal matrix, on the treatment of full-thickness cutaneous wounds.^47^ The AMSCs seeded on the acellular dermal matrix group led to a significant increase in the re-epithelialization rate and blood vessel density compared to that in the acellular dermal matrix only group.^47^ Moreover, AMSCs were found to co-localize and express VEGF after the transplantation, which suggests that AMSCs seeded on acellular dermal matrix can promote angiogenesis and accelerate the cutaneous wound healing process.^47^ The in vitro and in vivo differentiation of AMSCs into endothelial cells (ECs) have been reported in various studies.^11,48^ In hind-limb ischemia models, AMSCs were shown to successfully differentiate into ECs that contribute to neo-angiogenesis, suggesting the potential use of AMSCs as a source of endothelial cells for cellular pro-angiogenic therapies.^49^ The expression studies of angiogenic markers, after human AMSC transplantation in rat ECM, revealed a significant increase in the expression of VEGF and a higher volume of newly generated tissue enriched with vasculature compared to those in the group with ECM alone, indicating that AMSCs can promote tissue growth and angiogenesis.^50^ A comparative analysis of paracrine factor secretion between dermal sheath cells, BM-MSCs, and AMSCs revealed that insulin-like growth factor-1, VEGF-D, and IL-8 are highly expressed in AMSCs compared to that in BM-MSCs and dermal sheath cells.^51^ Moreover, results showed that VEGF-A and VEGF-D are major growth factors released by AMSCs that enhance the formation of new blood vessels indicating that adipose tissue is a preferred source of MSCs over other MSC sources to augment angiogenic-based therapeutic approaches.^51^

## Acceleration of wound closure

The therapeutic potential of AMSCs in the wound healing process owing to their ability to accelerate wound closure after skin injury has been demonstrated in multiple studies.^48,52,53^ Mesenchymal stem cells can stimulate dermal fibroblast migration and provide important signals for dermal fibroblast responses to cutaneous injury.^52^ Adipose-derived MSCs have been shown to significantly accelerate wound closure in normal and diabetic rats.^54^ The green fluorescent protein-labeled AMSCs implanted in wounds showed site-specific differentiation to epithelial and endothelial cells, which suggests that AMSCs can accelerate cutaneous wound closure and healing through differentiation, angiogenesis, and secretion of angiogenic factors, including VEGF.^54^ Another study demonstrated that self-assembled AMSC spheroids resulted in fast wound closure and angiogenic activity after their administration to the created wounds on rat dorsal skin.^55^ In a rat model, AMSCs were also shown to support topical skin adhesives to close wounds, indicating their potential use for wound closure.^56^ An in vivo study on diabetic and non-diabetic rats showed that local injection of AMSCs significantly accelerated wound closure in full thickness skin defects in both groups compared to that in the control.^57^ Adipose-derived MSC-based therapy has emerged as a new therapeutic strategy for the treatment of skin wounds. The transplantation of AMSC sheets into skin wounds in diabetic rats significantly accelerated wound closure and healing outcomes.^44^ Adipose-derived MSC treatment of dorsal full-thickness skin wound defects in a diabetic rodent model resulted in accelerated wound closure and healing compared to those in the control without AMSC implantation, suggesting that AMSCs engrafted into the local wound tissue enhance diabetic wound healing.^58^ Treatment with TNF-α in a rat excisional wound model resulted in enhanced wound closure and angiogenesis, and inhibition of IL-6 or IL-8 expression significantly delayed wound closure.^59^

## Re-epithelialization

During the wound healing process, macrophages secrete several growth factors and cytokines that induce the migration of fibroblasts, epithelial, and endothelial cells into the wound site, which promotes wound repair during the proliferative phase.^29,48^ Local implantation of autologous AMSCs significantly induced epithelialization, blood vessel density, and tissue granulation in full-thickness skin defects in diabetic and non-diabetic rat models.^57^ The AMSCs implanted in wounds showed site-specific differentiation to epithelial and endothelial cells, which indicates that AMSCs have the capacity to mediate cutaneous wound healing through differentiation, vasculogenesis and secretion of growth factors.^54^ Green fluorescent protein-labeled AMSCs in wounds showed localized differentiation to epithelial and endothelial cells at the wound site. The differentiation of AMSCs to epithelial cells was also reported in an in vitro study, which showed that all-trans retinoic acid treatment of AMSCs resulted in the formation of keratin fibers, and more than 80% of AMSCs were able to undergo epithelial differentiation.^60^ To examine whether AMSCs have the potential for wound repair, chronic wounds were created via clinical radiation to study the role of AMSCs in the treatment of chronic wounds.^61^ The findings of this study demonstrated that AMSC treatment resulted in observed re-epithelialization and the formation of new blood vessels.^61^ Results obtained from ex vivo experiments have shown that AMSCs enhance the proliferation and migration of dermal fibroblasts and keratinocytes. Thus, AMSCs contribute to re-epithelialization at the wound site via paracrine signaling to maintain the homeostasis of epidermis through the secretion of growth factors.^33,48^

## Immunomodulation of chronic inflammation

Platelet aggregation occurs immediately after injury to prevent blood loss, and different immune cells release cytokines and other factors that trigger an inflammatory response. Macrophages and neutrophils also secrete growth factors that promote repair of injured skin.^37,48^ Excessive inflammation during cutaneous wound repair has been linked to chronic wounds and scar formation.^31^ In chronic inflammation state, macrophages and neutrophils continuously release proinflammatory cytokines that upregulate the expression of MMPs, resulting in the degradation or inactivation of several important factors for wound repair.^29,37^ This chronic inflammation leads to further defects in the injured skin. The major therapeutic approaches for cutaneous wound repair aim to reduce excessive inflammatory responses in chronic injuries.

**Figure 3:**
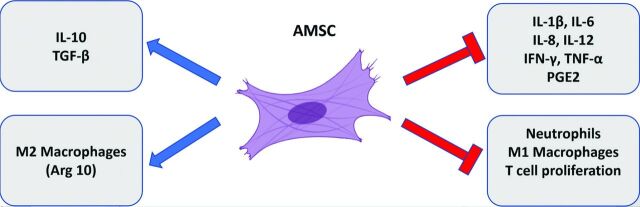
- Mesenchymal stem cells immunomodulatory roles in wound closure. Adipose-derived MSCs (AMSCs) interfere with the expression and secretion of various pro-inflammatory cytokines and selectively promote the expression of pro-healing and pro-fibrotic cytokines and cells. As well, AMSCs have a suppressive effect on inflammatory cells like neutrophils, M1 macrophages, and T cells. The cumulative effect of such roles of AMSCs is to shorten duration and enhance wound closure. TGF-β: transforming growth factor-β, PGE2: prostaglandin E2, IL: interleukin, TNF-α: tumor necrosis factor-α, IFN-γ: interferon gamma

The hypodermis layer is composed of adipocytes, AMSCs and blood vessels that secrete various factors involved in the regulation of inflammatory responses and new blood vessel formation.^62^ These therapeutic effects could be attributed to resident AMSCs, which secrete numerous anti-apoptotic signaling molecules and growth factors and contribute to the endogenous repair of wounds.^13,33,48^ Adipose-derived MSCs have the capacity to produce trophic mediators that modulate the inflammatory response in wounds and stimulate skin regeneration and repair (Figure 3).^33,48^ Numerous in vitro studies have shown that AMSCs possess immunosuppressive properties, as they secrete IFN-γ and prostaglandin E2.^63^ Adipose-derived MSCs can interact with various immune cells including T and B lymphocytes, NK cells, neutrophils, and dendritic cells.^33,48^ Another study showed that AMSCs express Jagged 1, which suppresses T-cell proliferation through Notch receptor pathway activation and NF-κB pathway inhibition.^64^ Furthermore, AMSCs have been reported to inhibit the secretion of proinflammatory cytokines from peripheral blood mononuclear cells (Figure 3).^65^ Interestingly, cells in lymphoid organs such as fibroblastic reticulocytes and lymphatic ECs stimulate the TNF-α and IFN-γ expression and activate T cells migration in lymph nodes, indicating a direct immunomodulatory role of the lymphatic cells during wound healing.^37,48^ macrophage, T lymphocytes, and platelets release TGF-β that induce fibroblasts to participate in this process. Moreover, the lymphatic cells have a key role in reducing oxygen tension and pH around the wound area, which results in the induction of angiogenesis via the release of FGF, VEGF, and TGF-β.^48^ Similarly, AMSCs can mediate angiogenesis, proliferation, and differentiation during the wound healing process via the secretion of various growth factors and cytokines.^66^

In murine models, administration of human AMSCs reduced inflammation via the inhibition of inflammatory cytokines expression and stimulation IL-10 expression.^67^ Treatment with TNF-α in a rat excisional wound model resulted in enhanced wound closure, angiogenesis, and infiltration of immune cells into the cutaneous wound.^59^ Inhibition of IL-6 or IL-8 expression significantly attenuated the TNF-α-stimulated wound closure and infiltration of immune cells, suggesting that the TNF-α-activated AMSCs enhance cutaneous wound repair through IL-6 and IL-8.^59^ Mesenchymal stem cells have been shown to upregulate anti-inflammatory cytokines such as IL-10 and IL-12 and downregulate pro-inflammatory cytokines such as IFN-γ, IL-1, and IL-6.^33^ Adipose-derived MSC transplantation into full thickness-skin grafts in a rat model resulted in a significant increase in skin graft survival rate compared to that in the control without AMSC infusion. Furthermore, the expression of IL-1β and TNF-α were significantly reduced, while that of IL-10 and Arg-1 were increased, indicating that the immunosuppressive properties of AMSCs can indirectly enhance skin graft survival.^68^ Several studies have reported the enhancement of cutaneous wound healing in after transplantation of AMSCs, suggesting the potential clinical applications of AMSCs for wound repair owing to their immunomodulatory effects during excessive inflammatory response in chronic cutaneous wounds.^33,48,69^

## Mesenchymal stem cell-derived exosomes in cutaneous regeneration

**Figure 4:**
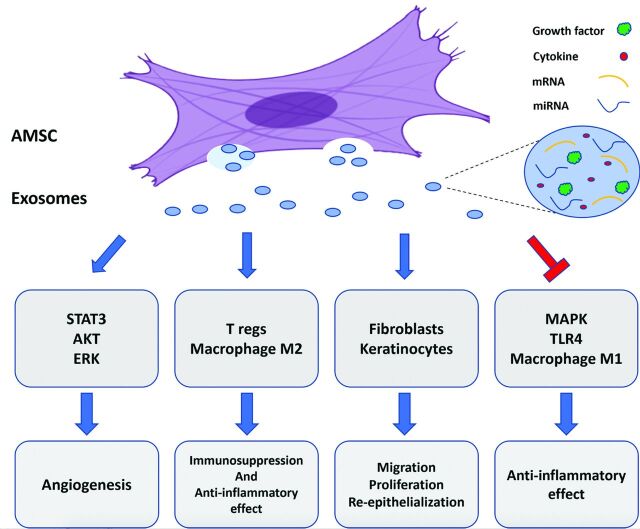
- Mesenchymal stem cell-derived exosomes regulate migration, proliferation, re-epithelialization, and angiogenesis, and have immunomodulatory roles at wound site. AMSC: adipose-derived mesenchymal stem cell, mRNA: messenger ribonucleic acid, miRNA: microRNA, STAT3: signal transducer and activator of transcription 3, AKT: protein kinase B, ERK: extracellular signal-regulated kinase, T regs: regulatory T cells, MAPK: mitogen-activated protein kinase, TLR4: toll-like receptor 4

Wound healing is a complex process that requires the involvement of several factors at the wound site, including, but not limited to, growth factors, cytokines, and ECM. Mesenchymal stem cells have been reported to play critical roles in all stages of cutaneous regeneration through paracrine signaling, which affects re-epithelialization, proliferation, and migration.^48,70^ Several studies have shown that MSC-derived exosomes can promote cutaneous regeneration through their effects on the inflammation, re-epithelialization, and remodeling phases (Figure 4).^71,72^ Exosomes are extracellular nanovesicles (50-100 nm) that encapsulates proteins, mRNAs, miRNAs, and soluble cytokines responsible for the paracrine effect of MSCs.^70-72^ Exosomes are secreted by a variety of cells and are found in body fluids such as blood and urine.^73^ The composition of MSC-derived exosomes varies from that of the exosomes released by other cell types, and some proteins can be used as markers to determine the identity of exosomes.^71^

Recently, there has been a growing interest in MSC-derived exosomes owing to their important role as modulators of the inflammatory response. The pro-inflammatory (M1) and anti-inflammatory (M2) phenotypes of macrophages play a key role in the cutaneous regeneration process. Adipose-derived MSC-derived exosomes were found to switch macrophage polarization toward the anti-inflammatory phenotype (M2).^74^ Among the miRNAs encapsulated in MSC-derived exosomes, miR-223 was found to suppress the pro-inflammatory pathway and enhance the anti-inflammatory response by reducing the production of IL-6; contrastingly, miR-199a plays a critical role in differentiation and re-epithelialization.^74^ Incubation of T cells with MSC-derived exosomes resulted in inhibition of T cell proliferation and promoted the switch toward Th17 polarization.^75^ Direct administration of MSC-derived extracellular vesicles effectively enhanced cutaneous regeneration by promoting dermal angiogenesis, migration, and re-epithelialization, and inhibited apoptosis in vitro via the downregulation of apoptosis-inducing factor (AIF) and upregulation of poly ADP ribose polymerase 1 (PARP-1).^69^ A recent study demonstrated that MSC-derived exosomes could downregulate pro-inflammatory cytokines such as IL-6, IL-1β, and TNF-α by suppressing MAPKs of ERK1/2, P38, and JNK.^76^ In addition, treatment of THP-1 cells with MSC-derived exosomes resulted in elevated expression of anti-inflammatory cytokines and polarization of macrophages toward M2 phenotype via inhibition of toll-like receptor 4 (TLR4) signaling by let-7 miRNA. Knockdown of AKT inhibited the immunomodulatory effect of MSC-derived exosomes on THP-1 cells indicating the important role of AKT activation via STAT3 signaling.^77^ Moreover, miRNA-181c carried by MSC-derived exosomes was reported to promote cutaneous regeneration by inhibiting TLR4 signaling.^78^ These studies indicate that MSC-derived extracellular vesicles can promote cutaneous regeneration and chronic wound healing through their effects on the inflammation, re-epithelialization, angiogenesis, cell migration, and ECM remodeling (Figure 4).

The bioactivities of MSC-derived exosomes seem to be controlled by wider range of miRNAs and proteins. Further investigations and studies are required to provide a comprehensive understanding of the key roles of MSC-derived extracellular vesicles in chronic wound healing and to facilitate their use as a therapeutic strategy in cutaneous regeneration and repair.

## Discussion and future perspectives

Adipose tissue is an easily accessible, abundant, and rich source of MSCs with a great therapeutic potential.^79,80^ The high MSC yield from adipose tissue, compared to that from BM, makes AMSCs an attractive cell source for the repair of chronic wounds and skin regeneration.^17^ Adipose-derived MSCs have been studied as an attractive alternative to BM-MSCs owing to their therapeutic potential for cutaneous wound repair. In pathophysiological conditions, the restoration of deep dermal layers is not efficient, which leads to either chronic wound or scar formation with loss of tissue function and structure.^26^ The treatment of chronic wounds is challenging because of our weak understanding of the underlying pathological mechanisms. Accordingly, new therapeutic strategies for the treatment of chronic wounds are required. Several therapeutic strategies based on AMSCs, including enhancement of angiogenesis, acceleration of wound closure, re-epithelialization, and immunomodulation of excessive inflammation, have been investigated for the treatment of chronic cutaneous wounds.

Various studies have shown that AMSC implantation into the wound site results in a significant increase in the formation of new blood vessels. Adipose-derived MSCs have been found to co-localize and secrete different mediators and growth factors, including VEGF, after transplantation, suggesting that they can promote angiogenesis and accelerate cutaneous wound healing process. Moreover, several reports have demonstrated that AMSCs have the capability to differentiate into ECs, suggesting their potential use in angiogenic-based therapeutic approaches. Adipose-derived MSCs have also been shown to significantly accelerate wound healing in normal and diabetic rats. The infusion of these cells can accelerate cutaneous wound closure and healing through differentiation, vasculogenesis and secretion of different mediators and growth factors. The AMSCs implanted in wounds show site-specific differentiation to epithelial cells, which suggests that they can enhance the cutaneous wound healing through differentiation to epithelial cells. In vitro experiments have shown that AMSCs can differentiate to epithelial cells, and more than 80% of AMSCs were able to undergo epithelial differentiation. Adipose-derived MSCs contribute to re-epithelialization at the wound site via a paracrine signaling to maintain the homeostasis of the epidermis. Furthermore, excessive inflammation during cutaneous wound repair has been linked to chronic wounds and scar formation.^38^ The major therapeutic approaches for cutaneous wound repair aim to reduce excessive inflammatory responses in chronic injuries. The AMSCs produce trophic mediators that modulate the inflammatory response in wounds and stimulate skin regeneration and repair. Moreover, AMSC implantation into the wound site reduces inflammation by inhibiting the expression of inflammatory cytokines and stimulating that of immunosuppressive cytokines including IL-10. The expression of IL-1β and TNF-α were significantly decreased, while that of IL-10 and Arg-1 were increased after AMSC transplantation into the wound site.

The wound microenvironment and resident cells, which secrete cytokines and growth factors, determine the capability of wound tissue repair. Systemic factors cause pathological changes to this microenvironment and play critical roles in non-healing wounds. These changes result in challenging conditions for cell-based therapies. Additionally, cutaneous chronic wounds are characterized by increased MMP activity, which result in degradation of growth factors and tissue lysis.^81,82^ Chronic wound fluid oxidative stress within the wound negatively affects the proliferation and migration activities of stem cells and represents local challenges to the therapeutic potential of MSCs for cutaneous wounds.^83,84^ Moreover, the number of cells required to achieve enhanced wound healing in patients is not well known. Novel strategies for cell delivery to maintain MSC functionality and survival are required to overcome these challenges. Recently, a few studies have shown that seeding AMSCs on a biomimetic hydrogel scaffold and biodegradable sutures, maintains the stemness of seeded cells, and induces wound healing process.^85,86^ Stromal vascular fraction (SVF) could be of great importance for its use as a scaffold for wound repair, as it comprises of microvasculature and ECM in addition to AMSCs that promote the healing process.

Prolonged culturing of AMSCs influences the viability of these cells due to replicative senescence.^87,88^ The senescent AMSCs lose their secretory repertoire and paracrine effects, which are pivotal for immunomodulation and angiogenesis during wound healing.^89^ The paracrine role of AMSCs at the wound site provides evidence of the relationships between these cells and other cellular components of the skin. While AMSCs secrete paracrine factors that promote skin wound healing, this role may also result in the progression of tumor cell division.^90^ Therefore, further studies using larger randomized controlled trials are required to ensure the safety of AMSC applications and elucidate the mechanisms underlying their efficacy.

In conclusion, AMSC-based therapy for non-healing or chronic cutaneous wounds in animal models has been shown to accelerate wound healing through different mechanisms. Therefore, the use of autologous AMSCs to promote cutaneous wound healing in patients appears to be a promising therapeutic strategy.
